# Ebola Virus Disease Outbreak — Nigeria, July–September 2014

**Published:** 2014-10-03

**Authors:** Faisal Shuaib, Rajni Gunnala, Emmanuel O. Musa, Frank J. Mahoney, Olukayode Oguntimehin, Patrick M. Nguku, Sara Beysolow Nyanti, Nancy Knight, Nasir Sani Gwarzo, Oni Idigbe, Abdulsalam Nasidi, John F. Vertefeuille

**Affiliations:** 1 Federal Ministry of Health, Federal Republic of Nigeria; 2 Center for Global Health, Global Immunization Division, CDC; 3 World Health Organization–Nigeria Office; 4 Lagos State Primary Health Care Board; 5 Nigeria Field Epidemiology and Laboratory Training Program; 6 UNICEF, Lagos Office; 7 Center for Global Health, Division of Global HIV/AIDS, South Africa Office, CDC; 8 Nigerian Institute for Medical Research

On July 20, 2014, an acutely ill traveler from Liberia arrived at the international airport in Lagos, Nigeria, and was confirmed to have Ebola virus disease (Ebola) after being admitted to a private hospital. This index patient potentially exposed 72 persons at the airport and the hospital. The Federal Ministry of Health, with guidance from the Nigeria Centre for Disease Control (NCDC), declared an Ebola emergency. Lagos, (pop. 21 million) is a regional hub for economic, industrial, and travel activities (1) and a setting where communicable diseases can be easily spread and transmission sustained. Therefore, implementing a rapid response using all available public health assets was the highest priority. On July 23, the Federal Ministry of Health, with the Lagos State government and international partners, activated an Ebola Incident Management Center as a precursor to the current Emergency Operations Center (EOC) to rapidly respond to this outbreak. The index patient died on July 25; as of September 24, there were 19 laboratory-confirmed Ebola cases and one probable case in two states, with 894 contacts identified and followed during the response. Eleven patients with laboratory-confirmed Ebola had been discharged, an additional patient was diagnosed at convalescent stage, and eight patients had died (seven with confirmed Ebola; one probable). The isolation wards were empty, and 891 (all but three) contacts had exited follow-up, with the remainder due to exit on October 2. No new cases had occurred since August 31, suggesting that the Ebola outbreak in Nigeria might be contained. The EOC, established quickly and using an Incident Management System (IMS) to coordinate the response and consolidate decision making, is largely credited with helping contain the Nigeria outbreak early. National public health emergency preparedness agencies in the region, including those involved in Ebola responses, should consider including the development of an EOC to improve the ability to rapidly respond to urgent public health threats.

## The Ebola Outbreak

The first known case of Ebola in Nigeria was in a traveler exposed in Liberia. On July 17, 2014, while under observation in a Monrovia, Liberia, hospital for possible Ebola, the patient developed a fever and, while symptomatic, left the hospital against medical advice. Despite advice against travel, on July 20 he flew by commercial airline from Monrovia via Accra, Ghana, to Lomé, Togo, then changed aircraft, and flew to Lagos. On arrival the afternoon of July 20, he was acutely ill and immediately transported to a private hospital where he was noted to have fever, vomiting, and diarrhea. During hospital admission, the patient was queried about Ebola and said he had no known exposure; he was initially treated for presumed malaria. Based on the patient’s failure to respond to malaria treatment and his travel from an Ebola-affected country in the region (2), treating physicians suspected Ebola. The patient was isolated and tested for Ebola virus infection while local public health authorities were alerted about a suspected case of Ebola. A blood specimen sent to Lagos University Teaching Hospital was confirmed positive for acute Ebola virus infection. The patient died on July 25.

Port Health Services conducted early contact tracing at the airport and worked with airlines and partners to ensure notification of the outbreak through International Health Regulations (IHR 2005) mechanisms (3). The EOC case-management team took over management of each laboratory-confirmed or suspected case, triaged potential patients, and decontaminated areas inhabited by them. Patients with suspected infection were isolated in the suspected case ward at the Ebola treatment facilities, initially in Lagos and subsequently in Port Harcourt. A contact tracing team staffed and supervised by skilled, dedicated epidemiologists was established to investigate all primary contacts and alert the case management team of symptomatic contacts for assessment and possible reclassification.* A suspected case† was reclassified as a confirmed case if reverse transcription–polymerase chain reaction (RT-PCR) detected Ebola virus in a blood specimen, and was ruled out if RT-PCR testing of two blood specimens collected at least 48 hours apart was negative. Additionally, testing for anti-Ebola virus immunoglobulin G, indicating an immune response to Ebola virus, was added to the testing protocol for PCR-negative suspected cases in persons with some symptoms who were epidemiologically linked to subsequent confirmed cases. When a contact became ill with a suspected case, the contact tracing team gathered data on persons exposed to that contact from the date of symptom onset in the event the suspected case should become laboratory confirmed. Having the capacity to conduct Ebola laboratory diagnosis in-country at the Lagos University Teaching Hospital facilitated rapid identification of confirmed cases and quick discharge of persons with suspected Ebola who tested Ebola negative.

**FIGURE 1:**
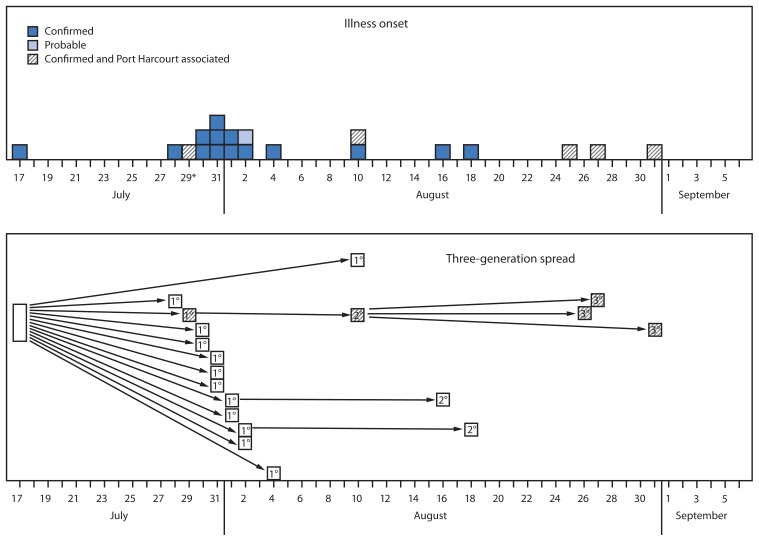
Number of cases of confirmed (n = 19) and probable (n = 1) Ebola virus disease, by date of illness onset and three-generation spread — Nigeria, July–August 2014 * The patient with July 29 illness onset was exposed in Lagos, traveled to Port Harcourt for treatment and initiated the Port Harcourt case cluster.

As of September 24, 19 laboratory-confirmed Ebola cases and one probable case had been identified (Figure 1). A total of 894 contacts were identified, and approximately 18,500 face-to-face visits were conducted by contact tracers to assess Ebola symptom development. Persons with suspected Ebola were transported to a suspected case isolation ward by the case management team, and persons who subsequently tested Ebola positive were moved to the confirmed case ward at the same facility in either Lagos or Port Harcourt. Eleven patients had been discharged, one additional patient had a confirmed diagnosis in the convalescent stage, and eight had died (seven confirmed; one probable) for an overall case fatality ratio of 40%. The isolation and treatment wards were empty, and 891 (all but three) contacts had successfully exited follow up. The remaining three contacts became ill but tested Ebola negative and were released from the isolation ward in Lagos. As is standard practice, upon release, the patients who had been suspected cases started a new 21-day follow-up as contacts because of the possibility that they were exposed in the ward. In this instance, no one was diagnosed with Ebola while these three contacts were in the ward, thus the likelihood of Ebola exposure was very low, and all three are due to exit follow-up on October 2.

Investigation of the index patient and all exposed contacts required coordination between multiple IMS response teams and across several cities in the course of the response. The three-generation spread of Ebola (all 19 confirmed and probable cases) to date can be traced to the index case through contact networks (Figure 1). Twelve of the 20 patients were exposed in two health facilities in Lagos. Four of the cases have been associated with a suspected case in a patient who traveled while ill via commercial aircraft from Lagos to Port Harcourt, Rivers State, and back (Figure 1). After the patient who traveled was discovered, manifests were collected from both flights, and attempts were made to contact passengers to ensure they had not become ill because >21 days had passed since the travel occurred. No ill or deceased passengers were identified. Overall, no new cases have occurred since August 18 in Lagos and August 31 in Port Harcourt, suggesting that the Ebola outbreak in Nigeria might have been contained (Figure 1).

## Public Health Response

The threat to Nigeria posed by the arrival in Lagos of a patient acutely ill with Ebola was potentially enormous. Lagos is Africa’s largest city and is also a transit hub for the region with air, land, and sea ports of entry (1). The dense population and overburdened infrastructure create an environment where diseases can be easily transmitted and transmission sustained. Suboptimal infection control practices in health centers lacking necessary equipment and supplies increase the risk for Ebola transmission to health care workers. Contact tracing efforts are burdened by the complex nature of transit, commercial, and public health notification and reporting mechanisms. The implementation of a rapid response that made use of the available public health assets was the highest priority at the onset of the outbreak, as was organizing the response using proven structures for the delivery of public health in Nigeria. To effectively address Ebola in this complex environment, the response was mounted quickly and used an IMS; both actions are largely credited with helping contain the outbreak early.

**FIGURE 2:**
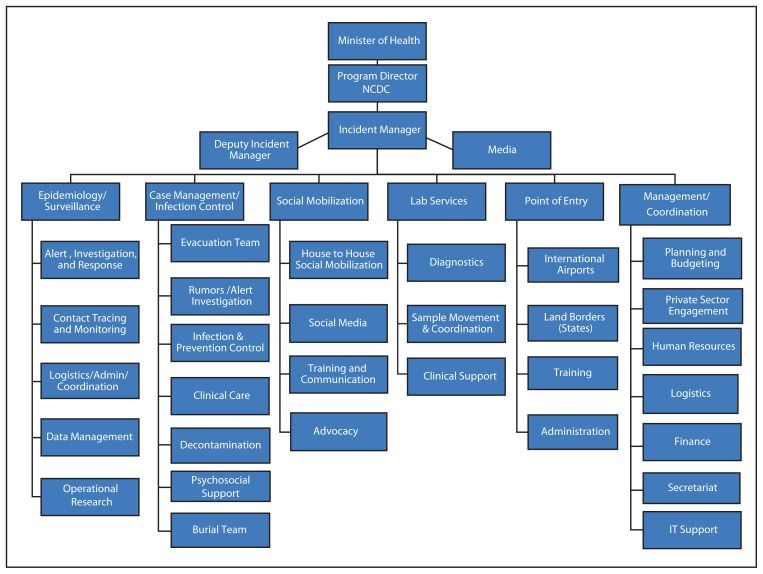
Organizational structure of the Ebola Response Incident Management Center — Nigeria, July–September 2014

Initially, NCDC and the Lagos State Ministry of Health established an Incident Management Center, which served as the overall implementing arm of the national response. The initial Incident Management Center was subsequently recast as the national EOC, in line with IMS nomenclature and national structures aimed at emergency response. The EOC expanded its operations to Rivers State when cases emerged there, and oversaw the monitoring of contacts in Enugu State with state health officials as part of the early outbreak response. There was a stated expectation that all partner organizations, donors, and response teams would work through the EOC structure, reporting to an Incident Manager (IM). In turn, the IM would be responsible to deliver accountable and transparent results to the NCDC and the federal Ministry of Health (Figure 2). The IM, responsible for oversight of the response, was selected based on IMS experience and competency rather than rank in government or public service.

Nigeria’s response benefited from the rapid use of its national public institution (i.e., NCDC), previous outbreak responses such as a major lead poisoning response in 2010, and its recent experience with polio eradication. In October 2012, responding to the declaration by the World Health Organization of polio eradication as a global public health emergency, and to improve its national response, the Government of Nigeria used the IMS to establish a national EOC as part of a new national emergency plan for the global polio eradication initiative (3). The use of IMS through the EOC changed the operational tempo, accountability measures, and programmatic success of the polio program. Indicators and dashboards (electronic displays of high level indicators for each response team monitored at the EOC) were developed to increase accountability of the program staff and spending. Through the EOC and the Nigeria Field Epidemiology and Laboratory Training Program (NFELTP) polio activities, state health system strengthening and preparedness was prioritized (4–6).

With the emerging Ebola outbreak, the Nigerian government moved quickly to enforce coordination of the national and state Ebola response efforts using the IMS/EOC structures and drew from its successful experiences. Specifically, the Ebola EOC IM was the polio EOC Deputy IM, and seeded the Ebola EOC with several secretariat and technical staff members from the National Polio EOC. Critical to demonstrating both national and state commitment, the Deputy IM was a senior member of the Lagos State Ministry of Health (Ebola was imported to Lagos State), with access to human and financial resources within the state health system. Immediately, the EOC developed a functional staff rhythm that facilitated information sharing, team accountability, and resource mobilization while attempting to minimize the distraction of teams from their highest priorities. An “Action Tracker” was developed that included specific tasks arising from each meeting, the person responsible, and the due date.

The overall design of the response rested within a senior strategy team made up of the IM, Deputy IM, and primary partner agencies (Doctors Without Borders, the United Nations Children’s Fund, the World Health Organization, and CDC). Six response teams were developed within the EOC specific to an Ebola response, including: 1) Epidemiology/Surveillance, 2) Case Management/Infection Control, 3) Social Mobilization, 4) Laboratory Services, 5) Point of Entry, and 6) Management/Coordination (Figure 2). Terms of reference and priority activities were developed by the strategy team to guide each operational team’s work; operational teams developed their own staffing, lists of material and financial needs, and a goal-oriented operational plan. The strategy group reviewed and approved all of the teams’ work and needed resources. Technical partners assigned staff throughout the operational teams in technical advisory roles aimed at building the capacity of the local teams and ensuring quality work.

What is already known on this topic?

The ongoing Ebola virus disease (Ebola) outbreak in West Africa has had an enormous negative impact on civil and public health systems in Liberia, Sierra Leone, and Guinea. Nigeria’s public health system includes a national public health institute (NCDC) and an Emergency Operations Center (EOC) and Incident Management System (IMS), created in 2012 when Nigeria declared polio a public health emergency and restructured its national polio program.

What is added by this report?

Applying lessons from its NCDC and successful polio EOC, Nigeria quickly established a National Ebola EOC after importation of the disease on July 20, 2014. The early use of the EOC/IMS system enabled the country to streamline a coordinated and effective response in Lagos, (pop. 21 million) and to expand that response to Port Harcourt, another large city. As of September 24, a total of 894 contacts in three states had been monitored, and 20 confirmed or probable Ebola cases identified, of whom eight died. No new cases had occurred since August 31, suggesting that the Ebola outbreak in Nigeria might have been contained.

What are the implications for public health practice?

African nations need to rapidly assess their readiness to manage the importation of Ebola. Preparedness activities could include planning EOC/IMS structures that can guide a coordinated and effective response to Ebola or any other public health threat. Where EOC already exists for other diseases like polio, such structures should be strengthened and used to mount effective responses to new threats like Ebola.

As an example of work planning efforts, the EOC Point of Entry team, led and staffed heavily from the Port Health Service, was responsible for identifying, listing, documenting, and risk-ranking of all the contacts of the index patient at the airport, including those on aircraft and those exposed during airport transit/handling of the index patient. Early in the response, this team mobilized to identify and track the index patient’s contacts in the airport and outside Nigeria. Port Health Service worked with airline and airport authorities and other stakeholders to gather information about contact passengers, decontaminate affected areas of the airport, and send a notice through the World Health Organization-International Health Regulations system to avoid possible spread of the disease. The Point of Entry team also established entry and exit screening at ports, which is being rolled out at additional ports and will continue for the duration of the regional outbreak to minimize the likelihood of either further importation or exportation of Ebola.

The Epidemiology/Surveillance team was responsible for contact tracing, operational research, management of alerts and rumors, and implementing community-based surveillance. For successful contact tracing, the Epidemiology/Surveillance management team included over a dozen trained, dedicated NFELTP, WHO, and CDC epidemiologists and was provided the target of listing all contacts of the index and subsequent Ebola cases in the response, and monitoring them in person daily to measure body temperature and check for the presence of other Ebola signs and symptoms (e.g., vomiting, diarrhea, and hemorrhage). In response, the team developed a staffing plan for Lagos that included over 150 contact tracers, vehicles, telephones, and mobile data platforms that the contact tracers could use to administer their questionnaires and report contact responses. In addition, the operational research arm of the Epidemiology/Surveillance team conducted a community Ebola assessment that informed training and communication efforts.

Directly linked to the contact tracing was the Social Mobilization strategy. This included teams of three social mobilizers who were trained and deployed to conduct house-to-house, in-person visits within specific radii of the homes of the Ebola contacts. For high-density areas, house-to-house teams covered a 500m radius, 1km in medium density areas and 2km for low density (7). As of September 24, approximately 26,000 households of persons living around Ebola contacts had been reached with house-to-house visits in Lagos and Rivers states.

Several issues were observed by the response team during Nigeria’s Ebola outbreak that could, in retrospect, have been mitigated through additional preparedness planning for public health emergencies. First, financial resources were slow to arrive at the EOC, a delay that threatened to impede the rapid expansion of containment activities across the response. Early activities were funded by the Lagos State government, international partners, and nongovernmental organizations. National preparedness efforts should consider how resources can be quickly accessible to fund the early stage of the response. Second, there were discrepancies among the levels of political leadership in fully appreciating the enormous consequences that even a small Ebola outbreak could have on civil institutions such as hospitals, airports, and public gatherings. Targeted education about the urgent need to fund, staff, and supply a response effort was provided to political leadership and should be considered for preparedness efforts elsewhere. Similarly, the Nigerian public did not have specific information about Ebola, and early information provided by the press, in advance of official information from the health authorities, was sometimes inaccurate and created a nationwide scare. This scare resulted in some persons resorting to extreme and sometimes harmful and ineffective measures to avoid infection such as consuming large quantities of salt water, even in places distant from the outbreak. Both issues could have been addressed through preparedness activities that focused on education and planning, as well as explaining Ebola to the public and describing how to respond should Ebola arrive in Nigeria. The Case Management team indicated that early efforts to establish an isolation ward were delayed due to a lack of Nigerian health care workers willing to care for patients with Ebola because of a lack of information and training about how to care for Ebola patients, and because care providers had been disproportionately impacted by Ebola in other affected countries. Preparedness activities should include orientation and training of physicians, nurses, and attendants to safely provide services with attention to infection control procedures and quality Ebola treatment at an appropriately designed facility. Another challenge was ensuring appropriate coordination of private sector engagement. The EOC system facilitated improved coordination through the designation of the Management and Coordination Team Lead as the private sector point of contact. Finally, some partners and parts of government were unfamiliar with the EOC/IMS system and its use as a means of streamlining coordination and response elements into one unified approach. The government-led EOC process could define opportunities for partners to place staff strategically in the national and local response efforts and could encourage this through the EOC response teams and management system. Further, EOC mechanisms should be tested through strategic exercises and use in non-Ebola responses.

Even with these identified challenges, Nigeria’s decision to use EOC/IMS to respond to Ebola resulted in a rapid, effective, and coordinated outbreak response. As of September 24, the Nigeria response had successfully limited the outbreak to 20 laboratory confirmed and probable cases (in two states) with the last cases occurring on August 18 and August 31 in Lagos and Port Harcourt, respectively. This limited spread and the rapid scale-up against the backdrop of the large, dense, urban environments of Lagos and Port Harcourt suggest early response efforts were successful; this is likely directly attributable to the Nigerian government’s strategic use of its public health institutions and the EOC/IMS structure to manage the response. The EOC/IMS approach should be a central part of national and subnational preparedness efforts for public health threats. EOC/IMS is a key component of the global health security agenda, along with Integrated Disease Surveillance and Response/International Health Regulations (IHR 2005).

## Footnotes

* An Ebola contact was defined as a person who had a known exposure to a confirmed, probable, or suspected case. Contacts were actively monitored for 21 days after the date of last exposure. The contacts were further classified by their exposure to the case as Type 1 (contact with body fluids such as blood, vomit, saliva, urine, or feces of a confirmed patient); Type 2 (direct physical contact with the body of a confirmed patient or decedent); Type 3 (contact with linens, clothes, or dishes/eating utensils); and Type 4 (a history of sleeping, eating, or spending time in the same household or room as a patient). Contacts were reclassified as suspected cases if they reported fever (or were observed to have temperature ≥99.5°F (37.5°C) axillary or ≥100.4°F (38.0°C) core and met one of the following criteria: 1) had vomiting, diarrhea, or bleeding from stool or mucous membranes; or 2) had two additional symptoms including headache, myalgia, arthralgia, or weakness.

† The case definition for a suspected case of Ebola in this outbreak was adapted from the World Health Organization recommended case definition (9). An illness in a patient who met all three of the following was a suspected case: a) Fever: The patient either reported having a fever, or if measured, had a temperature of ≥99.5°F (37.5°C) axillary or ≥100.4° (38.0°C) core; b) Exposure: The patient visited an affected area in the preceding 3 weeks or had contact with an ill person who visited an Ebola-affected area within 3 weeks of becoming ill; c) Presence of additional symptoms: The patient had any two of the following: bleeding (at mucous membranes or in stool), vomiting, diarrhea, headache, myalgia, arthralgia, or weakness. In addition, an illness was a suspected case if the patient met these two criteria: a) Fever: The patient either reported having a fever, or if measured, had a temperature of ≥99.5°F (37.5°C) axillary or ≥100.4° (38.0°C) core; b) Higher level exposure: Close contact with a confirmed Ebola case or with a person who died, if the person died from a febrile or unexplained illness and had visited an affected area within 3 weeks of becoming ill, or participation in a funeral within 3 weeks of having a fever in which 1) the funeral was conducted in an affected area, or 2) the deceased person had visited an affected area within 3 weeks of becoming ill (9). Suspected cases were confirmed to be Ebola by laboratory testing using RT-PCR to test blood for the presence of Ebola virus. In situations in which the suspected case was identified during a convalescent period, post-disease immunoglobulin G testing was conducted to assess an immune response to Ebola and/or semen samples were tested using RT-PCR for the presence of Ebola virus. Suspected cases were ruled out as confirmed if two consecutive negative RT-PCR tests spaced ≥48 hours apart were negative.
